# Case Report: Durable disease stability in a patient with MET exon 14 skipping mutation and brain metastasis NSCLC following radiotherapy and immunotherapy

**DOI:** 10.3389/fimmu.2026.1719029

**Published:** 2026-02-03

**Authors:** Ji Ma, Depeng Zhang, Yang Cui, Dongfang Meng, Zhigang Wei, Xin Ye

**Affiliations:** 1 Department of Oncology, Lung Cancer Center, The First Affiliated Hospital of Shandong First Medical University and Shandong Provincial Qianfoshan Hospital, Shandong Lung Cancer Institute, Jinan, China; 2 Department of Neurosurgery, People’s Hospital of Boxing, Binzhou, China; 3 Department of Anesthesiology, People’s Hospital of Boxing, Binzhou, China; 4 Department of Radiation Oncology, Shandong Cancer Hospital and Institute, Shandong First Medical University and Shandong Academy of Medical Science, Jinan, China; 5 Cheeloo College of Medicine, Shandong University, Jinan, Shandong, China

**Keywords:** brain metastasis, camrelizumab, lung cancer, MET exon 14 skipping mutation, radiotherapy

## Abstract

**Background:**

Non-small cell lung cancer (NSCLC) with brain metastasis (BM) is associated with poor prognosis. For those patients with MET exon 14 skipping mutation (METex14), although MET tyrosine kinase inhibitors (MET TKIs) have emerged, but their efficacy remains limited, with the median progression-free survival (PFS) no more than 14 months. Herein, we present a case of a NSCLC patient with BM and METex14, who achieved prolonged PFS of 41 months following brain radiotherapy initiation and camrelizumab (a PD-1 inhibitor).

**Case presentation:**

A 75-year-old man was diagnosed with lung adenocarcinoma with BM and METex14. He received 7 months of crizotinib as first-line therapy, after that, the lung and brain lesions enlarged. Then, programmed cell death-ligand 1 (PD-L1) showed the tumor proportion score (TPS) approximately 80%, he underwent brain radiotherapy combined with camrelizumab immunotherapy. After treatment, the lesions in the patient’s lung and brain were significantly reduced. Camrelizumab maintenance therapy was continued for 20 months until the appearance of pulmonary aspergillosis, with the patient achieving a PFS of 41 months.

**Conclusion:**

The combination of brain radiotherapy and camrelizumab demonstrated efficacy in a lung adenocarcinoma patient with BM and METex14. These findings suggest that immunotherapy may represent a potential treatment approach for high PD-L1 expression of METex14 NSCLC patients, warranting further investigation in larger cohorts.

## Introduction

Lung cancer is the most commonly diagnosed cancers and continues to be the leading cause of cancer mortality worldwide (1). Based on histological examination, it is broadly categorized into two main types: small cell lung cancer (SCLC) and non-small cell lung cancer (NSCLC), with the latter comprising approximately 85% of all diagnosed cases (2). NSCLC is further classified into three primary types: squamous-cell carcinoma, adenocarcinoma, and large-cell carcinoma. Among these, lung adenocarcinoma is the most common type, comprising around 40% of all NSCLC cases (3).

MET exon 14 skipping mutation (METex14) is one of the important oncogenic drivers in NSCLC and promotes tumor invasion, angiogenesis, and metastasis processes. This mutation, which can be found approximate 3% in non-squamous NSCLC cases, is a relatively rare driver gene-positive mutation in NSCLC (4). Brain metastasis (BM) is a common complication in NSCLC, approximately 40% of non-squamous NSCLC patients will eventually develop BM over the course of their disease (5). The median overall survival (OS) for NSCLC patients with BMs is 6 months, with 1-, 2-, and 3-year OS rates of 29.9%, 14.3%, and 8.4%, respectively (6).

Immune checkpoint inhibitors (ICIs) have revolutionized the treatment paradigm for advanced NSCLC, and first-line treatment with immunotherapy, with or without chemotherapy, improves overall survival (OS) and progression-free survival (PFS) compared with platinum-based chemotherapy (7). Camrelizumab, a humanized anti-programmed death receptor-1 (PD-1) monoclonal antibody, has demonstrated a trend toward improved intracranial PFS (median, 12.7 vs. 9.9 months; hazard ratio [HR], 0.45) and PFS (median, 9.7 vs. 6.7 months; HR, 0.57) versus with placebo in the first-line of NSCLC with BM (8).

Herein, we present a case of a patient diagnosed with METex14 lung adenocarcinoma with BM, who underwent targeted therapy, radiotherapy and immunotherapy. The patient achieved PFS of 41 months following radiotherapy initiation and immunotherapy.

## Case presentation

**Figure 1:**
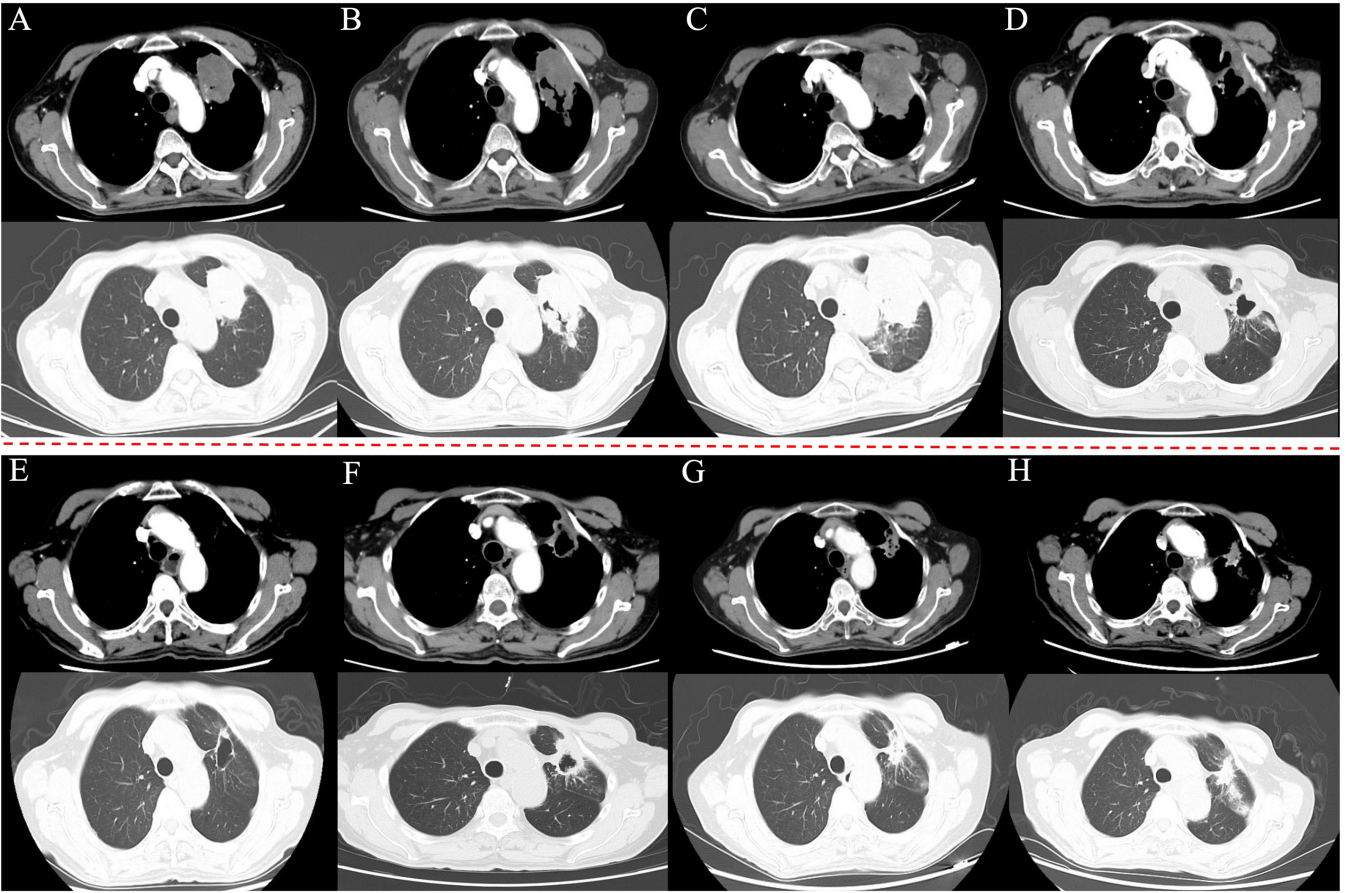
Chest CT scan of the patient during the treatment process at both the mediastinum window (above) and the lung window (below). **(A)** Initial imaging revealed a 5.7×4.2 cm mass of soft tissue-like density in the left upper lobe of the left lung. **(B)** After 2 months of crizotinib, the lung lesion (5.6×4.4 cm) assessed as stable disease. **(C)** After 7 months of crizotinib, the lung lesion size increased to 6.8×4.8 cm. **(D)** After two cycles of camrelizumab, the lung lesion size reduced to 3.6×3.0 cm. **(E)** After 18 months of camrelizumab maintenance, almost no tumors left in the mediastinal window. **(F)** After 20 months of camrelizumab maintenance, the lung lesion size increased to 4.2×3.0 cm. **(G)** After 2 months of voriconazole, the lung lesion size reduced to 3.0×2.4 cm. **(H)** At 41 months from WBRT initiation (with subsequent camrelizumab), the lung lesion (3.3×2.2 cm) assessed as stable disease (latest image).

**Figure 2:**
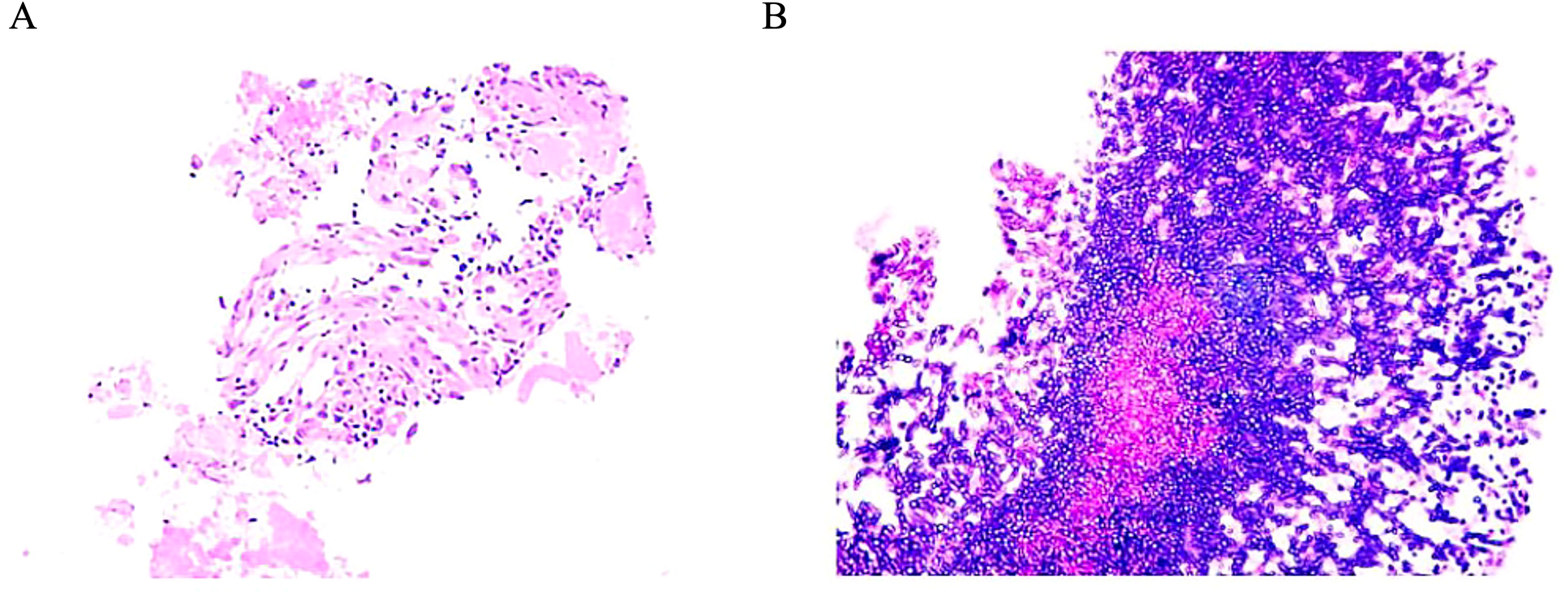
The pathology results of the lung lesion biopsy. **(A)** Initial biopsy of the lung lesion histologic was adenocarcinoma (100×). **(B)** After 20 months of camrelizumab maintenance, the second biopsy of the lung lesion was chronic inflammation of fibrous tissue and aspergillus infection (200×).

**Figure 3:**
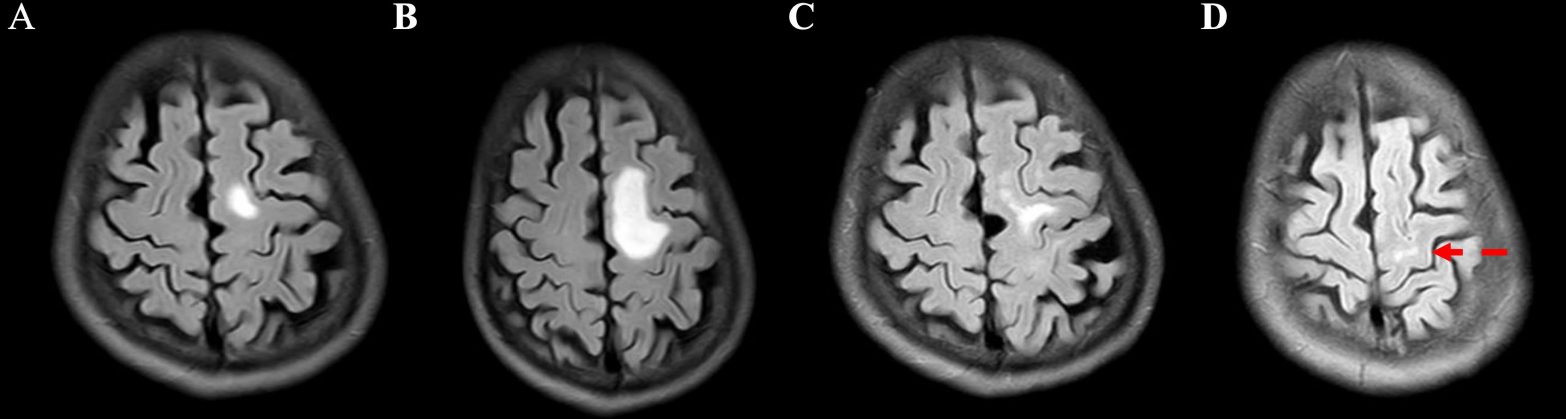
Brain MRI of the patient during the treatment process. **(A)** Initial imaging revealed a brain metastatic lesion about 1.2×1.0 cm in left frontal lobe. **(B)** After 7 months of crizotinib, the brain metastatic lesion size increased to 1.5×1.4 cm. **(C)** After radiotherapy and two cycles of camrelizumab, the brain metastatic lesion size reduced to 1.1×0.9 cm. **(D)** At 41 months from WBRT initiation (with subsequent camrelizumab), the brain metastatic lesion is almost undetectable (latest image).

A 75-year-old man presented with a cough and hemoptysis for one week. A chest computed tomography (CT) scan revealed a space-occupying lesion measuring 5.7×4.2 cm in the left upper lobe of the left lung (**Figure 1A**), with multiple swollen lymph nodes in the ipsilateral mediastinum, the largest measuring approximately 1.7 cm in diameter. The patient underwent puncture biopsy of the lesion. Immunohistochemical staining of the biopsy revealed positivity for CK, CK7, TTF-1, NapsinA, and P63, and negativity for CK5/6, CgA and Syn, the Ki-67 proliferation index was 5% (**Figure 2A**). Genetic testing revealed METex14 mutation (NGS, Geneseeq). Brain magnetic resonance imaging (MRI) revealed a metastatic lesion measuring 1.2×1.0 cm in left frontal lobe (**Figure 3A**). The patient was diagnosed with lung adenocarcinoma in the left upper lobe of the left lung at clinical stage IVA (T_3_N_2b_M_1b_).

The patient received a targeted therapy with crizotinib (250 mg p.o. bid) for 7 months. During this period, periodic imaging examinations assessed stable disease (SD, **Figure 1B**). Subsequently, a chest CT scan showed the lung lesion had increased to 6.8×4.8 cm (**Figure 1C**), and brain MRI revealed the BM had increased to 1.5×1.4 cm (**Figure 3B**). Programmed cell death-ligand 1 (PD-L1) testing revealed a tumor proportion score (TPS) of 80% (22C3, PD-L1 IHC 22C3 pharmDx™, formalin-fixed paraffin-embedded biopsy specimens). Whole brain radiotherapy (WBRT) was administered with 6 MV photons via bilateral opposed fields, with a total dose of 45 Gy (3 Gy/fraction, 5 fractions/week) and no additional boost, followed by camrelizumab (200mg on day 1, every 2 weeks) immunotherapy. After two cycles were completed, a chest CT scan showed a partial response (PR) in both the lung and BM lesions, with lesion sizes measuring 3.6×3.0 cm (**Figure 1D**) and 1.1×0.9 cm (**Figure 3C**), respectively.

**Figure 4:**
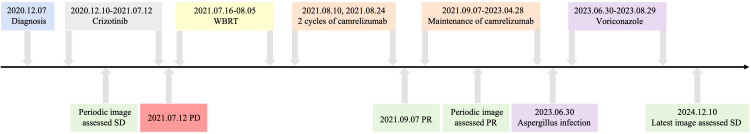
Timeline of the patient’s major treatment course and imaging evaluations since diagnosis. SD, stable disease; PD, progressive disease; WBRT, whole brain radiotherapy; PR, partial response.

Camrelizumab maintenance therapy was continued 20 months. During this period, regular imaging evaluations indicated PR (**Figure 1E**). Subsequently, a chest CT scan showed the lung lesion had increased to 4.2×3.0 cm (**Figure 1F**). Based on the CT evidence of progression, camrelizumab was discontinued and not restarted. A puncture biopsy of the lesion revealed chronic inflammation of fibrous tissue and aspergillus infection (**Figure 2B**). Voriconazole was administered for antifungal therapy. After 2 months, the chest CT showed the lung lesion size had reduced to 3.0×2.4 cm (**Figure 1G**). The patient continued regular reexaminations. The latest imaging examination, performed 41 months after initiating WBRT, showed stable lung lesion (**Figure 1H**) and an almost undetectable BM lesion (**Figure 3D**). In terms of adverse events, after WBRT, the patient experienced fatigue and mild headache, and immunotherapy-related grade 1 thoracoabdominal rash was observed. All adverse events were managed with supportive care, and no other toxicities were identified. The timeline of major treatments is presented in **Figure 4**.

## Discussion

This case report describes a 75-year-old male with BM and METex14 NSCLC who achieved a remarkable long-term PFS of 41 months following brain radiotherapy initiation and camrelizumab.

In the PROFILE 1001 trial, crizotinib was the first drug to demonstrate activity in METex14 NSCLC, achieving a median PFS of 7.3 months (9), which was superior to than that of first-line cisplatin/pemetrexed doublet chemotherapy (median, 4.8 months) (10). Subsequently, MET tyrosine kinase inhibitors (TKIs) emerged as promising therapies. In the VISION and GEOMETRY phase II trials, in patients with advanced METex14 NSCLC, tepotinib and capmatinib achieved median PFS of 12.45 months and 12.6 months, respectively (11, 12). Another MET inhibitor, savolitinib, approved in China, demonstrated a median PFS of 13.7 months (13). However, at the time of treatment, these medications were not yet available in China. Considering the patient’s old age and the relatively significant side effects associated with chemotherapy drugs, crizotinib therapy was selected as the first-line treatment option.

The efficacy of ICIs is closely correlated with the TPS of PD-L1. Elevated PD-L1 TPS levels are associated with longer PFS and OS in advanced NSCLC, with the greatest benefit in those patients with very high PD-L1 (≥ 90%) (14, 15). While MET TKIs are currently recommended as the first-line option for advanced METex14 NSCLC, patients with high PD-L1 expression may derive greater benefit from ICIs. A recent multicenter, retrospective, observational study compared outcomes between first-line MET TKI and ICIs ± chemotherapy in patients with METex14 NSCLC. The results demonstrated that for patients with a PD-L1 TPS ≥80%, first-line ICIs ± chemotherapy significantly prolonged PFS compared to MET TKI (median, 9.4 months vs. 5.0 months; HR, 0.50; *p* = 0.03). Among patients with BM, first-line MET TKI was associated with longer PFS (median, 11.70 months vs. 2.73 months; HR, 0.39; *p* = 0.02), and a lower percentage of intracranial progression (18.8% vs. 30.8%, *p* = 0.7) (16). However, the comparative efficacy of MET TKIs versus ICI regimens in BM patients has not been further explored based on PD-L1 expression levels. For patients with METex14 NSCLC and BM who exhibit high PD-L1 expression, treatment with ICIs may have a more favorable outcome compared to MET TKI.

The use of radiotherapy remains the cornerstone of BM management in NSCLC patients due to the low permeability of the blood-brain barrier (BBB) to most conventional anticancer drugs. WBRT has been the standard treatment for BM in NSCLC, improving BM control in 60-80% of patients. However, for oligometastatic disease, stereotactic radiosurgery (SRS) has emerged as the preferred standard of care, but it is associated with a higher risk of intracranial recurrence (17). The combination of radiotherapy and ICIs may have a potential antitumor synergistic effect. On the one hand, radiotherapy can induce immunogenic cell death in cancer cells, leading to the release of damage-associated molecular patterns. This process enhances neoantigens presentation and broadens the T-cell receptor repertoire (18). On the other hand, ICIs can activate peripheral T cells, facilitating their penetration into the BBB (19). Retrospective studies have shown that combination of ICIs with cranial radiotherapy provides a safe and tolerable treatment strategy for NSCLC patients with BM (20, 21). Although the patient had only a single brain metastasis, we ultimately selected WBRT instead of SRS based on the patient’s old age, considering that WBRT could provide prophylactic control of intracranial micrometastases and release more antigens to create a favorable tumor microenvironment for immunotherapy.

As patients with METex14 NSCLC are older than those with other oncogene-driven NSCLC, prioritization of effective and tolerable therapies is warranted in this population (22). For patients with advanced NSCLC and BM, the triplet therapy of chemotherapy, immunotherapy, and radiotherapy has shown encouraging efficacy. The CTONG2003 trial showed that, in first-line NSCLC with BM, triplet therapy (chemotherapy + camrelizumab + radiotherapy) improved both median intracranial PFS (19.1 vs. 9.9 months; HR, 0.42) and overall PFS (11.2 vs. 6.7 months; HR, 0.42) compared with chemotherapy plus radiotherapy (8). A case report of a patient with NSCLC and BM who achieved a complete response lasting 37 months following chemo-immunotherapy and radiotherapy (23). However, in advanced NSCLC patients aged ≥75 years, the chemo-immunotherapy combination may not improve survival compared with immunotherapy alone and may be associated with higher drug toxicity. A retrospective cohort study in Japan demonstrated that ICI-chemotherapy combination treatment did not improve survival and increased the incidence of grade 3 and higher immune-related adverse events compared with ICI monotherapy (24.3% vs. 17.9%, *p* = 0.03) in advanced NSCLC patients ≥75 years (24). Given the patient’s old age, chemotherapy was excluded from the treatment regimen; instead, camrelizumab monotherapy was selected as the second-line regimen, maintaining disease control for nearly 2 years until the appearance of pulmonary aspergillosis.

Immunotherapy-associated pulmonary aspergillosis is rarely reported in the literature. A case report of lung adenocarcinoma treated with 20 cycles of nivolumab (a PD-1 inhibitor) described significant tumor remission but acute progression of pulmonary aspergillosis (25).The activation of T-cell immunity may influence both tumor activity and the reactivation of T-cell mediated underlying conditions, particularly in older patients with weakened physical status, potentially induce them to aspergillus infection. In this case, the patient’s infections were successfully controlled with voriconazole treatment.

As a case report, this study has several limitations. First, a PET-CT scan was not performed following the initial pathologic diagnosis, primarily due to high cost and lack of insurance coverage, which may have resulted in the omission of micrometastases. Second, regular assessment of neurocognitive function should be routinely included into late toxicity monitoring following WBRT; however, formal neurocognitive testing was not performed in this patient. This omission precluded a comprehensive evaluation of long-term neurocognitive function following WBRT.

In conclusion, this case of METex14 lung adenocarcinoma with BM demonstrated prolonged PFS following brain radiotherapy initiation and immunotherapy. Prospective randomized trials are needed to validate the comparative efficacy of ICI treatment versus MET TKI therapy in METex14 NSCLC patients with BM and high PD-L1 expression. The occurrence of aspergillosis infection following immunotherapy need to be noticed.

## Data Availability

The original contributions presented in the study are included in the article/supplementary material. Further inquiries can be directed to the corresponding authors.
